# Recommendations for generating real-world evidence with regulatory-grade relevance and reliability

**DOI:** 10.1007/s10147-026-03160-6

**Published:** 2026-08-20

**Authors:** Yasutoshi Sakamoto, Hideaki Bando, Yoshihiro Aoyagi, Hiroki Sokuoka, Toshihiro Misumi, Koji Oba, Yukiko Mori, Jun Okui, Makoto Miyazaki, Yasunari Sadatsuki, Kazuma Koizumi, Rie Otake, Akinori Yuri, Eri Tajima, Taro Ueno, Takayuki Nakamura, Hiroki Yoshihara, Keisuke Itoya, Ryosuke Annaka, Harumasa Nakamura, Natsuko Okita, Hideaki Takahashi, Tomohiro Kuroda, Kenichi Nakamura, Takayuki Yoshino, Atsushi Ohtsu

**Affiliations:** 1 Translational Research Support Office, Division of Drug and Diagnostic Development Promotion, Department for the Promotion of Drug and Diagnostic Development, National Cancer Center Hospital East, 6-5-1 Kashiwanoha, Kashiwa, Chiba 277-8577 Japan; 2 Department of Gastroenterology and Gastrointestinal Oncology, National Cancer Center Hospital East, 6-5-1 Kashiwanoha, Kashiwa, Chiba 277-8577 Japan; 3 Department of Data Science, National Cancer Center Hospital East, 6-5-1 Kashiwanoha, Kashiwa, Chiba 277-8577 Japan; 4 Department of Medical Information, National Cancer Center Hospital East, 6-5-1 Kashiwanoha, Kashiwa, Chiba 277-8577 Japan; 5 Interfaculty Initiative in Information Studies, The University of Tokyo, Tokyo, 113-0033 Japan; 6 Center for Digital Transfomation of Healthcare, Kyoto University, Kyoto, 606-8501 Japan; 7 Department of Biostatistics, Keio University School of Medicine, Tokyo, 160-8582 Japan; 8 Pharmacovigilance Subcommittee, Drug Evaluation Committee, Japan Pharmaceutical Manufacturers Association, Tokyo, 103-0023 Japan; 9 Patient Safety Strategy BioPharma Department, Japan Patient Safety Division, R&D Japan, AstraZeneca K.K., Tokyo, 108-0023 Japan; 10 Integrated Evidence Generation Post-Marketing Surveillance, Bayer Yakuhin Ltd., Osaka, 530-0001 Japan; 11 Safety Program Operation, MSD.K.K, Tokyo, 102-8667 Japan; 12 Japan PMS & Re-Examination, Takeda Pharmaceutical Co., Ltd., Tokyo, 103-8668 Japan; 13 Safety Science 2 Department, Chugai Pharmaceutical Co., Ltd., Tokyo, 103-8324 Japan; 14 Flatiron Health, K.K., Tokyo, 108-0075 Japan; 15 SUSMED, Inc., Tokyo, 103-0023 Japan; 16 Nuclear Liability Office, Research and Development Bureau, Ministry of Education, Culture, Sports, Science and Technology (MEXT), Government of Japan, Tokyo, 100-8959 Japan; 17 Nishimura & Asahi (Gaikokuho Kyodo Jigyo), Tokyo, 100-8124 Japan; 18 Health Policy Bureau, Ministry of Health, Labour and Welfare, Tokyo, 100-8916 Japan; 19 Innovation Promotion Subcommittee, Pharmaceutical Industrial Policy Committee, Japan Pharmaceutical Manufacturers Association, Tokyo, 103-0023 Japan; 20 Department of Clinical Research Support, National Center of Neurology and Psychiatry, 4-1-1 Ogawahigashi-cho, Kodaira, Tokyo 187-8551 Japan; 21 Clinical Research Support Office, Research Management Division, National Cancer Center Hospital, Tokyo, 104-0045 Japan; 22 Regulatory Affairs Management Section, Clinical Research Planning Division, Clinical Research Support Office, National Cancer Center Hospital East, Chiba, 277-8577 Japan; 23 Division of Medical Information Technology and Administration Planning, Kyoto University Hospital, Kyoto, 606-8501 Japan; 24 Department of International Clinical Development, National Cancer Center Hospital, Tokyo, 104-0045 Japan; 25 Research Unit, Japanese Foundation For Cancer Research, Tokyo, 135-8550 Japan

**Keywords:** Real-World Data, Relevance, Reliability, Pharmacovigilance

## Abstract

Real-world data (RWD) and real-world evidence (RWE) are increasingly incorporated into regulatory decision-making to complement randomized controlled trials (RCTs), particularly in settings where conventional trial designs are infeasible, such as rare diseases, rare molecular subtypes, and post-marketing evaluation. Across regulatory authorities, the use of RWD/RWE is consistently framed by the fit-for-purpose principle, centered on two foundational concepts: relevance and reliability. However, the absence of operational guidance has created uncertainty regarding how academic registries and healthcare databases can be designed or upgraded to meet regulatory expectations. Drawing on practical experience with disease registries in Japan and their regulatory applications, this review proposes a purpose-oriented framework clarifying the levels of relevance and reliability required according to specific regulatory objectives. We categorize considerations into three use cases: (1) drug development for rare diseases and rare molecular subtypes; (2) Pharmacovigilance; and (3) evidence generation for clinical questions that cannot be sufficiently evaluated through randomized controlled trials (RCTs). For each category, we outline essential requirements related to study design, data elements, quality management, governance, statistical planning, and operational feasibility. We further compare regulatory expectations across the U.S. Food and Drug Administration (FDA), the European Medicines Agency (EMA), the Pharmaceuticals and Medical Devices Agency (PMDA), and the International Council for Harmonisation (ICH). While international convergence is evident at the level of principles, operational thresholds and implementation frameworks differ. By articulating relevance and reliability as a shared regulatory language and emphasizing purpose-driven design and early regulatory engagement, this review provides practical recommendations for generating regulatory-grade RWE that meaningfully informs regulatory decision-making while complementing conventional clinical trials.

## Background

According to the definition provided by the U.S. Food and Drug Administration (FDA), real-world data (RWD) refer to data relating to patients’ health status and/or the delivery of health care that are routinely collected from a variety of sources in real-world clinical settings, including electronic health records, medical claims data, and disease registries. Real-world evidence (RWE) is defined as the clinical evidence regarding the usage frequency, effectiveness, and safety of medical products that is derived from the analysis of such RWD [1].

Major applications of RWD/RWE include: (1) drug development for rare diseases and rare molecular subtypes; (2) Pharmacovigilance; and (3) evidence generation for clinical questions that cannot be sufficiently evaluated through randomized controlled trials (RCTs), such as those informing clinical practice guidelines. Although RCTs remain the core methodology for evidence generation, the use of RWD/RWE is becoming a realistic and increasingly important option in areas where patient populations are limited or where timely decision-making is required.

However, when RWD are used for regulatory decision-making, the mere availability of data is insufficient. It is necessary to carefully evaluate whether the data are appropriate for the intended research or regulatory purpose (***relevance***) and whether they possess sufficient quality to withstand regulatory assessment (***reliability***) [2, 3]. This concept has been articulated as the ***fit-for-purpose*** principle and is consistently emphasized across regulatory guidance issued by authorities including the FDA, the European Medicines Agency (EMA), and the Pharmaceuticals and Medical Devices Agency (PMDA). Furthermore, the International Council for Harmonisation (ICH) has clearly positioned relevance and reliability as the fundamental framework for regulatory use of RWD in ICH E6(R3) Annex 2 (Step 2 draft, 2024) [3].

Despite increasing regulatory guidance, practical interpretation of relevance and reliability remains heterogeneous across stakeholders, particularly for academic-led registries and healthcare databases that were not originally designed for regulatory use. This gap between principle-based guidance and operational implementation has created uncertainty regarding how to design, govern, and evaluate RWD infrastructures intended for potential regulatory applications.

In this set of recommendations, drawing primarily on examples from disease registries in Japan and their use in regulatory contexts, we aim to clarify the levels of relevance and reliability required for three categories of data: (1) Data to Support Drug Development and Regulatory Submissions; (2) Data to Support Pharmacovigilance and Safety Evaluation; and (3) Data to Support Other Forms of RWE (Including Evidence for Clinical Practice Guidelines).

## Overview of relevance and reliability requirements for regulatory use of RWD across regions

Guidance on the regulatory use of RWD has been issued by the FDA, EMA, and PMDA, while ICH E6(R3) Annex 2 (Step 2 draft, 2024) provides an overarching international framework. A common principle across these documents is the fit-for-purpose approach, whereby relevance—alignment of population, exposure, outcomes, and covariates with the intended objective—and reliability of RWD are evaluated in accordance with the specific research or regulatory purposes.

With respect to reliability, all regions emphasize three core elements—accuracy, completeness, and traceability—and highlight the importance of ensuring transparency in data origin, collection and management processes, and data processing and analysis. In addition, when RWD are used as external control data, ensuring comparability of target populations and endpoints, and minimizing confounding and bias through appropriate study design, are consistently identified as critical considerations. Regardless of the specific disease area, early consultation with regulatory authorities and the establishment of documented operational frameworks are also uniformly recommended.

At the same time, each regulatory authority demonstrates distinctive characteristics in its guidance. ICH E6(R3) Annex 2 Draft guidance presents principle-based, source-agnostic concepts and serves as a higher-level framework that underpins regional guidance [3, 4]. PMDA guidance is characterized by detailed, practice-oriented requirements, including contractual arrangements with registry holders, access to source data, and the preparation of standard operating procedures and records [5, 6]. FDA guidance provides source-specific evaluation frameworks for registries, electronic health records, medical claims data, and externally controlled trials, with detailed considerations related to variable definitions, missing data, bias, and timing of data capture [7–11]. In 2024, the EMA published a Reflection Paper that organizes principles for the use of RWD in non-interventional studies, positioning current efforts as a step toward future institutionalization (Table 1) [12].

**Table 1:** Comparative overview of relevance and reliability requirements for regulatory use of RWD

Item	Japan (PMDA)[5, 6]	United States (FDA) [7–11]	Europe (EMA) [12]	ICH E6(R3) Annex 2 [3, 4]
Key documents	Basic Principles on Use of Registries for Applications; Points to Consider for Ensuring Reliability; RWD Reliability Q&A	RWD-related guidance (EHR, registries, external controls, etc.)	Reflection Paper on RWD (2024)	E6(R3) Annex 2 Draft guidance (2024) (General principles for safety assessment supplemented by ICH M14)
Definition and scope of RWD	Real-world data generated in healthcare settings, including registries and EHRs	Medical records, claims data, registries	Healthcare-related data used in observational studies	Diverse data sources generated in routine clinical practice
Relevance	Ensuring comprehensive and comparable capture of population, exposure, outcomes, and covariates aligned with study objectives; emphasis on external control comparability; confirmation of governance and transparency	Validity and comparability of population, exposure, and outcomes according to purpose; alignment of observation periods and variable definitions	Representativeness, measurability, availability, granularity of endpoints; appropriateness relative to research objectives (fit-for-purpose)	Availability of data elements aligned with purpose; prespecification in protocols; proportionate quality management; appropriate consent and ethical compliance
Reliability	Data quality (accuracy, completeness, consistency); SOPs and records; access to source data; system validation	Accuracy, completeness, traceability; explicit QA/QC; clarity and validation of variable definitions; documentation of data lineage (e.g., ICD-10, CPT)	Consistency and completeness of data collection; transparency of data origin and processing; third-party verifiability	Reliability assessed by accuracy, completeness, and traceability under fit-for-purpose principle; accountability for data provenance and processing
Early regulatory consultation	Strongly recommended through PMDA face-to-face consultations (registry use, use planning, reliability consultation)	Recommended prior to IND/NDA/BLA submissions	Emphasis on early scientific advice	Explicitly states desirability of prior consultation with regulatory authorities
Relationship to study design	Purpose-specific use of RWD (external controls, clinical outcomes, natural history); attention to comparability and selection bias	Comparability required for externally controlled and observational studies	Bias and confounding minimization required	Application of Quality by Design approach
Transparency and accountability	Disclosure of registry governance, funding, and access policies	Documentation of data structure and definitions in submission materials	Disclosure of research objectives, data definitions, and limitations	Design and records ensuring reproducibility and verifiability

BLA = Biologics License Application; CPT = Current Procedural Terminology; E6(R3) = ICH Harmonised Guideline for Good Clinical Practice (Revision 3); EHR = Electronic Health Records; EMA = European Medicines Agency; FDA = U.S. Food and Drug Administration; ICH = International Council for Harmonisation of Technical Requirements for Pharmaceuticals for Human Use; ICD-10 = International Classification of Diseases, 10th Revision; IND = Investigational New Drug Application; M14 = ICH Guideline on General Principles on Plan, Design and Analysis of Pharmacoepidemiologic Studies that Utilize Real-World Data for Safety Assessment of Medicines; NDA = New Drug Application; PMDA = Pharmaceuticals and Medical Devices Agency; QA/QC = Quality Assurance/Quality Control; RWD = Real-World Data; SOP = Standard Operating Procedure

## Purpose-specific considerations for relevance and reliability

### Data to support regulatory submissions

Based on accumulated experience with the RWD and RWE in regulatory submissions, this section organizes key considerations related to relevance and reliability and proposes approaches to database construction that reflect practical realities in clinical settings. The relevance and reliability discussed here assume that the data will be used as part of regulatory submission materials and that regulatory authorities may review individual patient-level data.

**Fig. 1:**
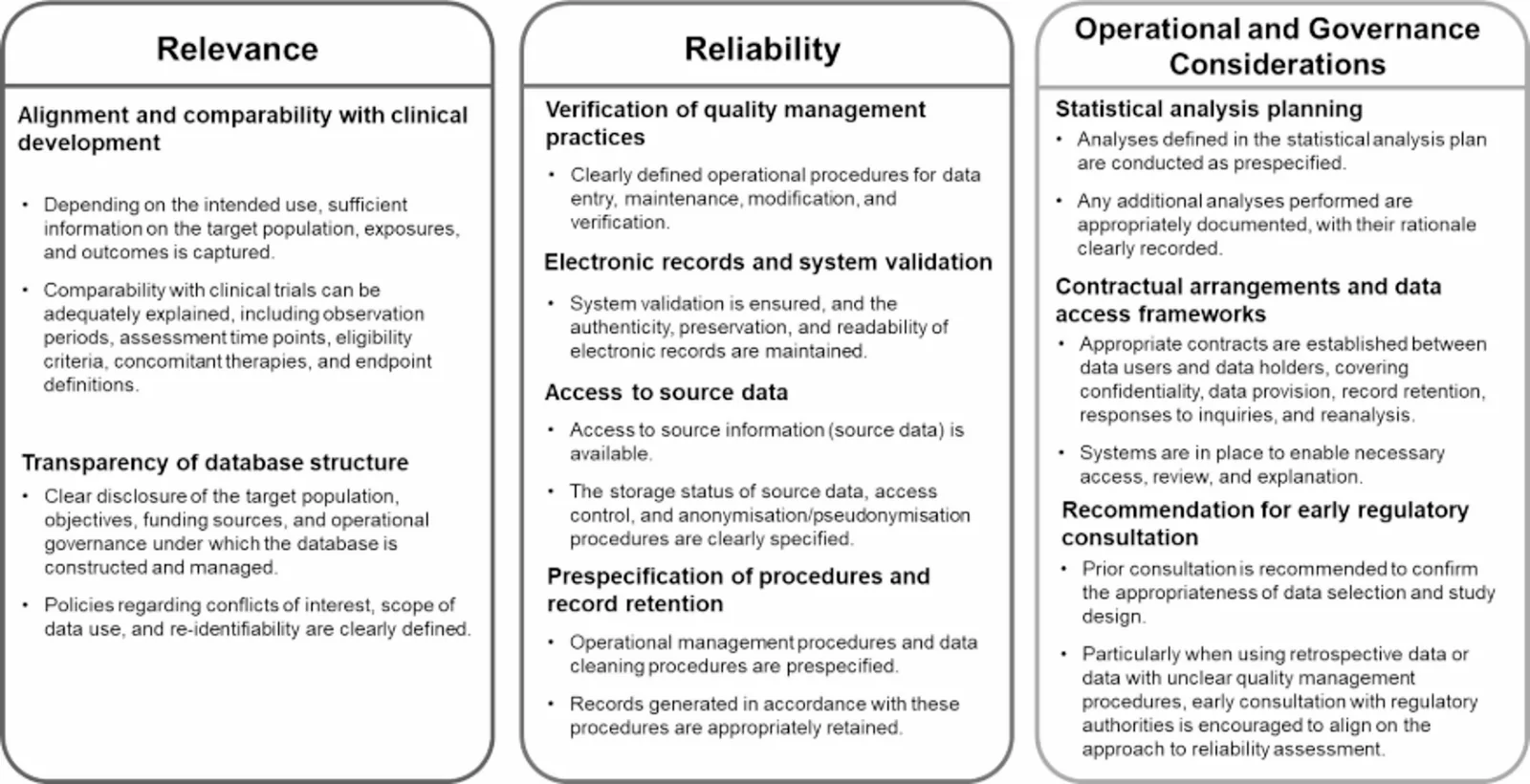
Key considerations for generating real-world data to support drug and medical product development are organized according to the concepts of *relevance*, *reliability*, and other operational considerations. Relevance focuses on alignment with clinical development objectives and ensuring comparability with clinical trials, as well as transparency of database structure and governance. Reliability addresses the establishment and documentation of quality management practices, electronic record management and system validation, access to source data, and prespecification of procedures with appropriate record retention. Other considerations include statistical analysis planning, contractual and data access frameworks, and the importance of early consultation with regulatory authorities, particularly when retrospective data or data with limited quality management information are used

From this perspective, the key considerations for constructing such data can be consolidated into nine practical points: two related to relevance, four related to reliability, and three related to other operational considerations, as outlined below (Fig. 1).

### Relevance

It is essential that clinicians and healthcare professionals with expertise in the relevant development field clearly define, based on practical experience in clinical development, the data elements that should be collected. If the data are intended to be used as key evidence for regulatory purposes, data collection should, in principle, be conducted through prospective observational studies. This is because certain data elements that are critical for regulatory evaluation—such as key baseline characteristics, timing of assessments, and information related to patient consent—are difficult to collect retrospectively. Accordingly, data should be designed to ensure that necessary information is collected without excess or insufficiency. In addition, it is desirable to conduct exhaustive case registration that captures the full range of clinically anticipated patient profiles for the disease of interest.

i. Alignment and comparability with clinical development

For cases included in databases or registries, eligibility criteria should be defined in alignment with future clinical trials. When RWD are used as external control data, differences in patient populations and baseline characteristics can have a substantial impact on analytical results. Therefore, baseline factors that are expected to influence treatment effects or prognosis—many of which overlap with variables routinely collected in clinical trials—should be comprehensively captured. These data are essential for appropriate adjustment of confounding factors during analysis.

Primary, secondary, and exploratory endpoints should also be collected using assessment methods and definitions consistent with those employed in clinical trials. Such alignment should be carefully considered at the design stage.

When conducting prospective observational studies, it is necessary to establish systems that allow assessments to be performed at time points aligned with the observation periods and evaluation schedules used in clinical trials. Misalignment of time axes is difficult to correct retrospectively and therefore requires particular attention during study planning.

In comparative analyses, when toxicities associated with standard clinical practice or concomitant therapies are expected to act as significant confounding factors, such information should be prospectively included among the data elements to be collected.

ii. Transparency of database structure

Databases and registries are often constructed and operated by academic institutions. From an ethical perspective, it is practical to conduct data collection as academic research that has undergone ethical review in accordance with national ethical guidelines for life science and medical research involving human subjects.

Within this framework, it is desirable that research objectives, target populations, funding sources, and governance structures are clearly described in study protocols and publicly available documents. Appropriate systems should also be established to identify, manage, and disclose conflicts of interest within the academic research framework.

When data are collected through prospective observational studies and used for regulatory purposes, it is necessary to obtain consent for such use in accordance with the latest ethical guidelines, with explicit disclosure in the consent documents that regulatory authorities may review the data.

### Reliability

It is important to establish reliability assurance systems that are sufficient for regulatory use while taking into account available personnel and resources. Reliability is evaluated not only in terms of data quality—centered on accuracy, completeness, and traceability—but also based on whether the procedures and records necessary to ensure such quality are appropriately established and maintained.

i. Verification of Quality Management Practices

Quality management procedures used in clinical trials and interventional studies can serve as a reference when developing quality management systems for observational research, while allowing for reasonable simplification appropriate to the study design and objectives. From the perspective of regulatory use, standard operating procedures and implementation records related to data entry, maintenance, revision, and verification should be prepared in advance.

When resources are limited, a stepwise approach—prioritizing essential procedures—may be acceptable, provided that the rationale is clearly documented. Essential processes necessary to ensure data reliability, including change histories and correction records generated during data collection and management, should be systematically recorded and retained.

ii. Electronic records and system validation

For RWD collected and stored electronically, system validation consistent with the principles of Good Clinical Practice (GCP), Good Vigilance Practice (GVP) and Good Post-marketing Study Practice (GPSP) is required. Although many electronic data capture (EDC) systems commonly used in clinical research are considered to meet these requirements, it is important, at the time of system implementation, to confirm whether vendors maintain GCP/GVP-compliant systems and supporting documentation.

iii. Access to Source Data

When RWD/RWE are used as part of regulatory submission materials, systems must be established to allow access to source data during regulatory review and reliability inspections. In particular, when RWD are intended for use as external control data, individual patient-level data should be prepared under the assumption that consistency with source documents will be verified, in a manner comparable to clinical trial data.

If cases intended for regulatory use are predefined, it is desirable to implement monitoring and source data verification (SDV) using a risk-based approach from the case accrual stage. When cases are selected for regulatory use after data collection has been completed, it is operationally reasonable to limit monitoring and SDV to the selected cases during dataset preparation. Even when cases are not intended for regulatory submission, monitoring activities—such as verification that data entry procedures and informed consent processes are appropriately conducted—may still be valuable, provided that sufficient resources and budget are available.

iv. Prespecification of Procedures and Record Retention

Data cleaning procedures should be clearly prespecified prior to implementation, and records demonstrating adherence to these procedures should be retained. When procedural or methodological changes occur during registry operation or data analysis, the details, rationale, and timing of such changes should be appropriately documented.

## Operational and governance considerations

i. Statistical Analysis Planning
ii. When RWD/RWE are used for regulatory submissions, it is critically important to analyze data using objective and reproducible methods. In particular, when external control data are used, careful consideration must be given to data characteristics and potential sources of bias to ensure scientific validity. Regulatory guidance on externally controlled trials emphasizes the importance of prespecifying statistical analysis plans, clearly identifying confounding factors, ensuring consistency in the definition of the index date and observation periods, aligning observation periods and assessment methods, and appropriately handling missing data at the planning stage. In addition, selection of comparable populations, appropriate confounding adjustment, and assessment of potential biases should be ensured [13].
iii. Contractual Arrangements and Data Access Frameworks
  Because regulatory submissions are typically made by pharmaceutical companies, contractual arrangements are required when academic-held registries are used. Such contracts should address confidentiality, data provision, record retention, response to regulatory inquiries, and reanalysis requirements. In addition, systems should be established to enable data access and explanation, as necessary, to support regulatory reliability assessments.
iv. Recommendation for Early Regulatory Consultation
  At the registry construction stage, it is recommended to utilize regulatory consultation frameworks to obtain advice on governance structures and data elements. Furthermore, consultation regarding registry use plans is desirable to confirm the appropriateness of intended use and the adequacy of evaluation items. Once specific use cases are identified, it is important to engage in further consultation to confirm that relevance and reliability appropriate to the intended purpose have been ensured.

Many healthcare-related databases and registries in Japan have been constructed and operated under constraints of limited budgets and personnel. Therefore, it is not realistic to implement all measures under constraints of limited budgets and personnel. Consequently, it is essential to allocate scarce resources to critical elements and prioritize quality assurance activities. Accordingly, it is necessary to prioritize elements related to relevance—such as data items and timing of collection—and reliability—such as preparation of procedures, record retention, and monitoring. Importantly, the rationale for the selected level of implementation should be clearly explainable. Early consultation with regulatory authorities is important for both database holders and users of healthcare-related data to clearly understand the standards required for their intended use and to identify any gaps in advance. This allows database holders to consider measures to address any deficiencies, and enables users to make early decisions regarding the feasibility of data utilization.

## Data to support pharmacovigilance and safety evaluation

**Fig. 2:**
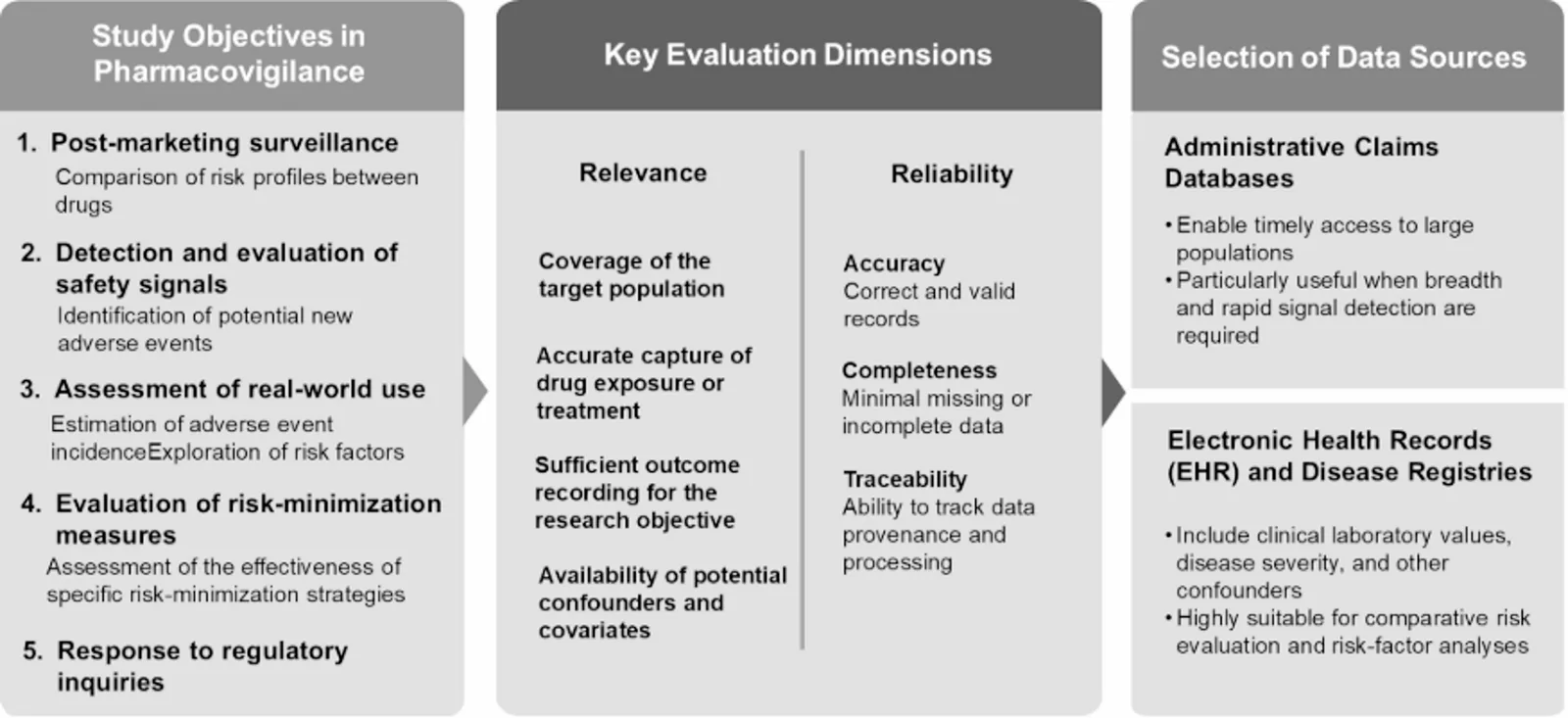
Conceptual framework for selecting real-world data sources in pharmacovigilance studies. Study objectives such as post-marketing surveillance, signal detection, and evaluation of risk-minimization measures determine the requirements for real-world data. These requirements can be assessed using two key dimensions, **relevance** (fitness of the data for the research objective) and **reliability** (data quality and traceability). Depending on these considerations, appropriate data sources such as administrative claims databases or electronic health records and disease registries may be selected

The World Health Organization defines pharmacovigilance (PV) as “the science and activities relating to the detection, assessment, understanding and prevention of adverse effects or any other medicine/vaccine related problem.” [14]. In PV, a wide range of RWD are utilized according to purpose, including post-marketing surveillance, detection and evaluation of safety signals, assessment of the effectiveness of risk minimization measures, and responses to regulatory inquiries (Fig. 2).

The ICH M14 guideline, General Principles on Planning, Designing, Analysing, and Reporting of Non-interventional Studies That Utilise Real World Data for Safety Assessment of Medicines, explicitly states that generation of robust evidence to be used for regulatory purposes depends on the reliability and relevance of the data and the application of sound methods to analyse such data [4].

### Relevance

In PV, the relevance of RWD is evaluated based on the data source limitation in relation to the specific objectives. Objectives in PV are diverse and can be broadly categorized as follows:

- Comparative risk assessment between medicinal products for specific adverse events
- Estimation of incidence rates and exploration of risk factors to understand real-world use
- As part of safety signal assessment, ‘signal detection,’ which involves rapidly and comprehensively exploring the potential association between a medicinal product and previously unknown adverse events, and ‘signal evaluation,’ which examines the clinical details of the detected events and their potential relationship to the drug.
- Assessment of the effectiveness of risk minimization measures
- Responses to inquiries from regulatory authorities

It is difficult for a single data source to satisfy all of these objectives. Therefore, selection of data sources requires a thorough understanding of their characteristics and limitations. For example, administrative claims databases, which cover large populations and allow for feature regularly updated new information, are often suitable for safety signal detection, where breadth and timeliness are prioritized. In contrast, when the objective is comparative risk assessment or exploration of risk factors, EHR data or disease registries—which may capture clinical laboratory values, disease severity, and other potential confounders—often provide higher relevance.

ICH M14 identifies representativeness of the target population, adequate sample size, and the availability of necessary exposure, outcome, and covariate information as key elements in assessing relevance [4]. When no single data source fully meets the research objective, it is necessary to clearly define which aspects of relevance are prioritized and to reflect this prioritization in study design and interpretation.

### Reliability

In ICH M14, reliability is defined as a concept encompassing accuracy, completeness, and traceability of the data, as well as the ability to explain data provenance and processing.

When RWD are used for regulatory purposes, PMDA requires that the reliability of the data can be appropriately explained. Where necessary, it is desirable to seek prior advice through consultation frameworks focused on registry or database reliability.

The principles related to quality management, electronic record management, access to source data, and preparation of procedures and records are fundamentally common to those described in ‘Data to Support Drug Development and Regulatory Submissions’. These considerations are equally important in PV. In particular, transparency and reproducibility in the processes of data extraction, transformation, and analysis form the foundation for regulatory assessment of reliability.

### Other considerations

i. Use of Registries
  Registries can serve as useful data sources in PV, as they allow for predefined endpoint definitions and follow-up procedures based on study protocols, thereby supporting a certain level of data quality. There are examples of registries being used for post-marketing surveillance including all-case surveillance. However, because registries may be focused on disease-specific information, they may lack data necessary to construct appropriate comparator groups. This limitation should be carefully considered when registries are used for comparative safety evaluations.
ii. Use of Databases in Post-Marketing Surveillance
  In Japan, database studies conducted as part of post-marketing surveillance represent the most widely utilized form of RWD use in PV. However, when target populations or outcomes cannot be appropriately defined within a given database, implementation of such studies may be difficult from the perspective of relevance. To avoid such situations, it is important to utilize regulatory consultation frameworks at the planning stage and to engage in thorough discussion with regulatory authorities regarding the suitability of data sources and the appropriateness of study designs.

## Data to support other forms of RWE (Including evidence for clinical practice guidelines)

Even when real-world evidence is not intended for direct use in regulatory submissions or Pharmacovigilance measures, the concepts of relevance and reliability remain important when evidence is generated in relation to specific research objectives. This section summarizes minimum considerations for prospective database construction and key points for interpreting existing databases when generating RWE to inform clinical practice guidelines and similar purposes.

### Relevance and reliability in database construction

#### Relevance

Even when regulatory use is not anticipated, the concept of relevance articulated by regulatory authorities is useful in general database design. To enable clinically meaningful comparisons and interpretation, it is desirable to organize target populations, endpoints, observational designs, and confounding factors in advance, and to define data elements that are neither excessive nor insufficient, including items that are difficult to collect retrospectively.

While comprehensive collection of baseline characteristics allows for sensitivity analyses with adjustment for background factors, increasing the number of data elements also increases operational burden at clinical sites. Therefore, it is important to define the minimum necessary data elements in accordance with study objectives and to consider phased expansion where feasible, taking implementation practicality into account.

With respect to endpoint definitions, it is desirable to refer to representative and widely accepted definitions used in clinical trials and interventional studies, with consideration for future comparability and reproducibility. In prospective observational studies, the selection of assessment time points and observation periods can influence analytical results; thus, realistic specifications should be established while balancing feasibility and the required level of relevance.

#### Reliability

It is important to establish rational reliability assurance systems that are appropriate to available personnel and resources. Even simplified systems can increase the potential for future data utilization if procedures and records related to data entry, maintenance, revision, and verification are appropriately prepared and retained.

With respect to missing data, investigators should understand the causes of missingness and its potential impact on analytical results, and should assess robustness through sensitivity analyses where appropriate. For electronic data collection, systems that allow tracking of modification histories and audit trails are desirable.

Although access to source data and implementation of monitoring or source data verification (SDV) are not mandatory when regulatory use is not anticipated, it is practically advisable to retain the ability to respond retrospectively to such requirements if future utilization becomes necessary.

### Key considerations when interpreting existing databases

When reflecting RWE in clinical practice guidelines or similar outputs, it is essential to appropriately interpret existing databases based on a thorough understanding of their characteristics. Major data sources used for RWE generation include administrative claims data (receipt and Diagnosis Procedure Combination [DPC] data), electronic health record (EHR) data, and registry data. These data sources differ substantially in structure, coverage, and granularity of available information.

When such databases are used for secondary analysis, it is necessary to understand the original purpose for which the data were collected and the processes by which they were generated and managed, and to evaluate their relevance and reliability in relation to the research objective. In this review, key considerations are organized in Table 2 from the perspectives of relevance—such as alignment with clinical development objectives, comparability, and transparency of database structure—and reliability, including accuracy, completeness, consistency, and traceability [15].

**Table 2:** Key considerations when interpreting existing databases

Data source	Considerations for relevance	Considerations for reliability
Administrative claims data (receipts, DPC)	The underlying population differs according to the type and size of insurers, limiting representativeness and generalizability DPC data are limited to acute inpatient care and originate only from institutions participating in the DPC system, thereby restricting patient populations and facilities Diagnoses may reflect insurance claim diagnoses rather than clinical diagnoses Laboratory results and detailed outcome information are generally unavailable; outcomes often need to be inferred from combinations of medical procedures and prescription data, requiring outcome validation	Standardized coding systems (e.g., ICD) are used, providing a certain level of accuracy Claim practices and coding procedures may vary across institutions and personnel. Because claims data are generated for reimbursement purposes, clinical detail and consistency are not necessarily ensured DPC data are generated consistently based on diagnosis group classifications and bundled payment rules; however, data characteristics may change due to system revisions or changes in reimbursement rules and operational practices Interpretation requires clarification of the applicable healthcare systems and reimbursement rules during the study period, as well as ensuring traceability by documenting temporal changes and their potential impact
Electronic health record (EHR) data	When the number of participating institutions is small, patient characteristics and prescribed therapies may be biased Even with a large number of institutions, data may be skewed toward specific types of hospitals (e.g., academic centers, community hospitals) or regions Information management policies and security practices differ across institutions, and departmental systems are often operated in a fragmented manner, resulting in incomplete centralization of required data Differences in EHR vendors and system configurations may lead to variation in master code systems or insufficient use of standardized codes.- Available data elements vary depending on the database holder Unstructured data, including free-text entries, require additional processing to enable analysis Linking data across institutions is difficult when patients receive care at multiple facilities	Data provider governance structures and data quality assurance systems should be confirmed Classification bias may arise during structuring and extraction of unstructured data; validation of methods and documentation are essential Methods and scope of data cleansing and validation differ across databases and may affect accuracy and consistency Mortality information is generally limited to in-hospital deaths or deaths recognized within the institution.- Accurate ascertainment of survival outcomes requires linkage with other public data sources, such as regional or national cancer registries Missing data and their potential impact should be evaluated, and robustness of results should be assessed through sensitivity analyses where appropriate Ensuring transparency and traceability regarding who processed the data, when, and how, during extraction, transformation, and processing is particularly important
Registry data	Patient characteristics may be biased depending on registration criteria.- Participation is limited to patients who have provided consent, introducing selection bias toward more cooperative patients When registries are conducted in parallel with clinical trials, they may include a higher proportion of patients who are ineligible for trial participation Governance structures and data quality assurance systems of registry operators should be confirmed	Data entry procedures differ across registries, and accuracy depends on procedures and adherence The presence or absence of data cleaning, monitoring, and audit systems has a substantial impact on reliability Inadequate update frequency or follow-up rates can reduce reliability

## Discussions

The regulatory use of RWD has the potential to transform drug development and evaluation frameworks by enabling the translation of clinical research findings into real-world medical practice. Drawing on registry-based experiences in oncology drug development in Japan, including ctDNA-guided trials and external control evaluations, we highlight the practical challenges encountered when aligning academic data infrastructures with regulatory expectations. To realize this potential, it is essential not only to understand the concepts of relevance and reliability—consistently emphasized by regulatory authorities—but also to operationalize them according to specific regulatory purposes. In this context, relevance and reliability should be understood as purpose-dependent constructs rather than fixed technical standards.

Importantly, the required intensity of relevance and reliability varies across use cases. Data supporting regulatory submissions and external control comparisons demand the highest level of documentation, traceability, and source verification. PV activities require robust accountability and transparency while accommodating diverse safety objectives. In contrast, other forms of RWE, such as evidence informing clinical practice guidelines, may rely on proportionate quality assurance mechanisms, provided that methodological robustness and interpretability are ensured. Recognizing this gradient of requirements is essential for efficient and scientifically sound data infrastructure design. We propose a gradient model of evidentiary intensity for regulatory-grade RWD infrastructure, in which the required depth of documentation, degree of traceability, level of source verification, and robustness of governance increase in proportion to the regulatory consequence of the intended use. Under this model, data supporting marketing authorization or label expansion represent the highest evidentiary tier, whereas PV activities and other non-submission RWE occupy intermediate or proportionate tiers. This conceptualization helps clarify that relevance and reliability are not binary attributes, but scalable requirements aligned with regulatory impact.

Within this gradient framework, relevance and reliability function as scalable constructs rather than fixed technical thresholds. Relevance refers to whether the target population, exposures, outcomes, and covariates are appropriately defined in relation to the research objective, whereas reliability denotes whether data accuracy, completeness, traceability, and provenance are adequately ensured. These concepts function not merely as technical criteria but as a shared regulatory language that facilitates dialogue between academic investigators, industry sponsors, and regulatory authorities. Early engagement with regulatory authorities should therefore be considered a structural component of fit-for-purpose database design rather than an optional procedural step.

Academic registries and healthcare databases, many of which were originally established for clinical research or quality improvement, increasingly face expectations for regulatory applicability. To respond to this shift, such infrastructures must evolve from research repositories into governance-transparent, documentation-ready, and potentially regulatory-grade platforms. Even when immediate regulatory use is not anticipated, embedding proportionate procedures for documentation, data provenance, and upgrade potential can enhance long-term utility.

Historically, randomized controlled trials (RCTs), beginning with the streptomycin trial in pulmonary tuberculosis, established the methodological foundation for regulatory science [16]. While RCTs remain indispensable nearly eight decades later, the increasing complexity of modern medicine—including rare diseases, molecularly fractionated subpopulations, and time-sensitive therapeutic innovation—has exposed structural limitations of traditional trial paradigms. In this context, RWD and RWE should not be viewed as alternatives to RCTs but as complementary evidentiary modalities that can be strategically integrated within a coherent regulatory framework.

Direct integration and aggregation of data from EHR across multiple healthcare institutions into a single database has also attracted attention as an approach. However, in Japan, substantial barriers to realizing this approach remain, including the lack of progress in EHR standardization and the difficulty of obtaining institutional approval to provide medical record information to external parties. At the same time, as the government promotes the development of infrastructure for EHR information-sharing services, the adoption of standards such as FHIR for EHR is expected to be further encouraged going forward, which in turn is expected to facilitate the integration and collection of data from EHR.

Looking ahead, continued technological advancement, refinement of regulatory systems, and international harmonization are expected to promote the generation and utilization of RWD/RWE that meet standards of relevance and reliability. A purpose-driven, governance-transparent, and consultation-oriented ecosystem for RWD will be essential to establish a scientifically robust and ethically sustainable foundation for evidence generation, ultimately supporting regulatory decision-making and improving patient care. Establishing internationally harmonized principles for relevance and reliability will be essential to enable the responsible integration of RWD and RWE into regulatory science while preserving the scientific rigor historically ensured by randomized trials.
